# Iranian Journal of Basic Medical Sciences, comparison of the three recent years

**DOI:** 10.22038/IJBMS.2021.19401

**Published:** 2022-01

**Authors:** Bizhan Malaekeh-Nikouei, Leila Arabi, Ali Roohbakhsh, BiBi Sedigheh Fazly Bazzaz

**Affiliations:** 1 Nanotechnology Research Center, Pharmaceutical Technology Institute, Mashhad University of Medical Sciences, Mashhad, Iran (Assistant Editor); 2 Pharmaceutical Research Center, Pharmaceutical Technology Institute, Mashhad University of Medical Sciences, Mashhad, Iran (Assistant Editor); 3 Biotechnology Research Center, Pharmaceutical Technology Institute, Mashhad University of Medical Sciences, Mashhad, Iran (Editor–in–Chief)

Greetings of the Season, best wishes for a Merry Christmas and a wonderful year ahead, and thanks for all your support throughout the year. We take this opportunity to give you a brief update regarding the current state of the journal.

Human society is still in the grips of a COVID-19 pandemic. Although we are experiencing a better situation after worldwide vaccination, new variants of the virus are the main problem. Certainly, this situation has an impact on both scientific research and publication. In this editorial, we aimed to highlight different aspects of Iranian Journal of Basic Medical Sciences (IJBMS) metrics including the trends of CiteScore and impact factor, analysis of submitted and accepted manuscripts, etc. 

**Figure 1:**
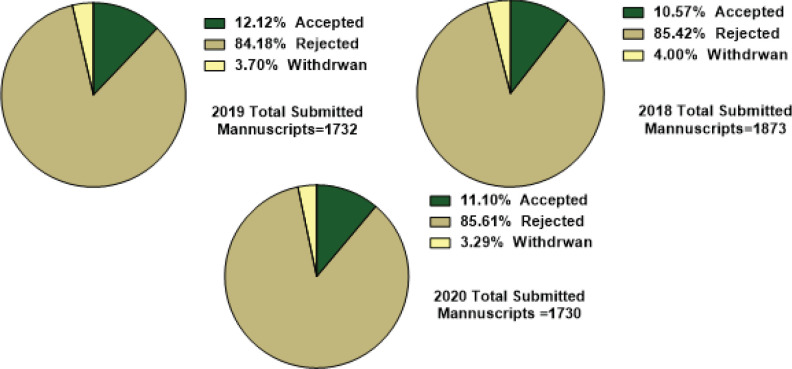
Analysis of accepted, rejected, and withdrawn manuscripts from 2018 to 2020

The percentages of submitted, rejected, and accepted manuscripts in 2018, 2019, and 2020 have been presented in Figure 1. Almost, the same trend was observed in the number of total submissions and rejected and accepted manuscripts in the recent three years. 

**Figure 2:**
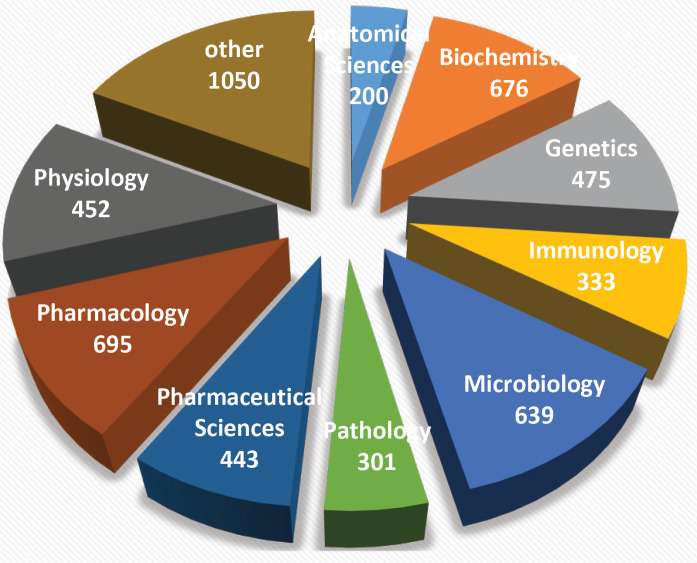
Subject of manuscripts submitted to Iranian Journal of Basic Medical Sciences from 2018 to 2020

The subjects of manuscripts submitted to IJBMS in the three recent years were summarized in Figure 2. IJBMS received manuscripts in different subjects such as biochemistry, anatomical sciences, genetics, microbiology, pharmaceutical sciences, physiology, immunology, pathology, and pharmacology. The most submitted manuscripts were in the fields of pharmacology and microbiology.

In 2019 and 2020, the number of published articles was increased from 15 to 17 in each issue. About 90% of published articles were original (Table 1). IJBMS encourages all scientists to send review manuscripts with hot topics to present their updated research results. 

**Figure 3:**
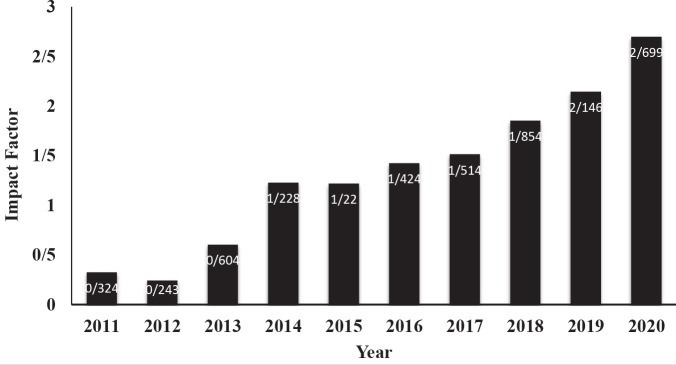
Trend of Iranian Journal of Basic Medical Sciences impact factor

**Figure 4.**
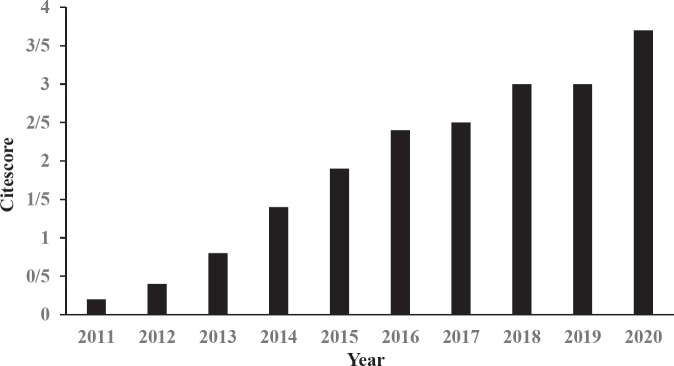
Trend of Iranian Journal of Basic Medical Sciences CiteScore

Figure 3 shows the impact factor changes of IJBMS issued by Clarivate Analytics (ISI). IJBMS impact factor showed an uptrend. This factor was changed from 1.854 in 2018 to 2.699 in 2020. The same trend was observed in CiteScore (Figure 4). CiteScore calculation is based on Scopus data in a 4-year window.

Some modifications were done in the journal’s website in the guide for authors section including conflict-of-interest, publication ethics, and article retraction based on Committee on Publication Ethics (COPE) guidelines. The FAQ section was updated and the author’s contributions statement was added for manuscript submission. Although we face some problems in the IJBMS editorial office such as prolongation of the review process, simultaneous submission of manuscripts to different journals, withdrawal after submission, submission of manuscripts by unrelated persons, etc., a better situation will be expected following implementation of COPE guidelines.

This editorial is a good chance to thank all our colleagues, associate editors, reviewers, and the authors whose contributions are what really make a journal great. And we thank the editorial office and the the publishers for their competence and efficiency. *Happy New Year*!

**Table 1:** Type of published articles in Iranian Journal of Basic Medical Sciences in the three recent years (2018–2020)

**Year**	**Original article**	**Short communication**	**Review article**	**Mini-review**	**Letter to editor**
**2020**	182	4	17	1	1
**2019**	166	11	21	1	0
**2018**	162	9	12	0	0

